# Cerebro-Spinal Meningitis, or “Spotted Fever”

**Published:** 1872-04

**Authors:** 


### Editorial Department.

### Cerebro-Spinal Meningitis or "Spotted Fever."

ANSWER TO CORRESPONDENTS.

The epidemic of cerebro-spinal meningitis which was spoken of in our last journal, as having recently visited Buffalo for the first time, continues to prevail with unabated frequency; the health officer's report for March, shows thirty two deaths from this disease alone. As we have already said, we are unable to throw any great light upon the nature or causes of this disease, but we would faithfully record the facts and circumstances of its appearance, and direct attention to the importance of studying the conditions and surroundings of all cases as they appear, thus hoping to finally gain some better and more satisfactory knowledge of its nature, causes and modes of cure. With this view we have noted the general hygienic conditions in the forty cases which have already fallen under our own care, or been seen in connection with our professional friends. These furnish representative cases of the disease as described by all authors, some mild in character and terminating favorably, others of active or malignant form and terminating fatally. It is impossible to determine at present, the proportion of deaths to cases, our own statistics show nothing, on this point, being called to see many of the cases because severe and certain to end fatally. It seems probable that the death rate is not so great as has generally been estimated in most other places where it has prevailed epidemically. Of the forty cases twelve are now known to have ended fatally, and of the remaining, others are likely to also end fatally, however, taking away the cases seen in consultation, mainly on account of their severity, and the proportion would be lessened nearly one half.

We are asked, is it contagious? For ourselves we answer no, and we believe the profession with remarkable unanimity, have regarded it as non-communicable by the sick to the well. Its prevalence in the French garrisons and barracks without extending to the civil population, and some other isolated facts giving the appearance of its extending by contagion led a few physicians to suppose it "infectious" or contagions, but we believe that their views were not sustained by subsequent observation, and that it may safely be regarded as settled, that it is not contagious. Again, we are asked, is it malarious? or does it arise from sewers and other sources of atmospheric contamination? In Buffalo, its rise and progress are connected, so far as can be known, with no animal or vegetable putrifaction, nor with any miasmatic or other deleterious or offensive emanations. It has made its appearance in the hovels of the poor and palaces of the rich; it has been about equally fatal when seen in the most salubrious and best ventillated apartments as when occurring in places destitute of all healthy surroundings. We cannot answer this question better than by quoting from the excellent work of Stille, upon Epidemic meningitis which we would recommend to all who desire to study this disease. Speaking of the causes he says: "It may be said in general terms that epidemic meningitis has occurred in all portions of the temperate zone, inhabited by European races and their decendants, in all sorts of localities, among all ranks and conditions of society, at all ages, and in both sexes, and that it is therefore in the strongest sense of the word a pandemic disease. And, when it is added that the disease is not disseminated by contagion, it evidently falls in to the category of the diseases referred to, of whose occurrence no other explanation is at present possible than that they are directly produced by a specific atmospheric poison."

Does it resemble typhus fever, and is it a new phase of that disease? Every experienced physieian when he meets his first case, though he may not recognize its true nature, will at once see that he has a group of remarkable symptoms and that it is something he has never before seen; he will soon decide upon its true character by exclusion, rejecting every other form of disease, he will soon arrive at the true nature of the malady. The cases do not resemble anything we have ever before seen, the symptoms, appearance of patient, and history of attack, are quite unlike that present in any other disease. Still, there are some resemblances to other low forms of disease, and some physicians liken it to typhus. We cannot better point out the wide distinction, than by quoting a tabular summary made by Stille.

| EPIDEMIC MENINGITIS. | TYPHUS FEVER. |
| --- | --- |
| A *pandemic* disease. Occurs in places remote from one another, and without intercommunication. | Essentially an *endemic* disease. Always due to local causes. Spreads by intercommunication only. |
| Attacks all classes of society. Is never primarily developed by squalor and deficient ventilation. | Attacks primarily the poor, filthy, and crowded, alone. |
| Is not contagious. | Contagious in a high degree. |
| More males than females attacked. | The two sexes are equally affected. |
| More young persons than adults. | More adults than young persons. |
| Generally occurs in winter. | Epidemics are irrespective of season. |
| Eruptions are wanting in at least half of the cases ; they occur within the first day or two. | The eruption is rarely absent, and appears between the fourth and the seventh day. |
| The eruptions are very various, including erythema, roseola,' urticaria, herpes, &c. Ecchymoses are common. | The eruption is uniformly roseolous and then petechial. Ecchymoses are rare. |
| Headache acute, agonizing, tensive. | Headache dull and heavy. |
| Delirium often absent; often hysterical, sometimes vivacious, sometimes maniacal. Generally begins on the first or second day. | Rarely absent; usually muttering. Rarely begins before the end of the first week. |
| Pulse very often not above the natural standard; often preternaturally frequent or infrequent. Is subject to sudden and great variations. | A slow pulse exceedingly rare. Its rate is pretty constantly between 90 and 120. |
| "The temperature is lower than that recorded in any other typhoid or inflammatory disease" It is also very fluctuating. | The temperature is always more or less elevated, and it does not fall until the close of the disease. "The skin is hot, burning, and pungent to the feel " |
| The body has no peculiar smell. | The mouse-like odor of typhus is characteristic. |
| The tongue is generally moist and soft ; sordes of the teeth, &c. is rare. | The tongue is generally dry, hard and brown ; and the teeth and gums fuliginous. |
| Vomiting, generally of billious matter, is an almost constant and urgent symptom, especially in the first stage. | Vomiting is rare, and not urgent. |
| Pains in the spine and limbs of a sharp and lancinating character are usual, and evidently neuralgic. | Pains are dull, heavy, and apparently muscular. |
| Tetanic spasms in a very large proportion of cases and within the first two or three days. They are due to an inflammatory exudation within the spinal canal. | Tetanic spasms are unknown in typhus. Convulsions sometimes occur, due to "pyæmia." |
| Cutaneous hyperæsthesia is a prominent symptom. | The sensibility of the skin is generally blunted. |
| Strabismus common. | Rare. |
| The eye, if injected, has a light red or pinkish color. | The blood in the conjunctival vessels has a dark hue. |
| The pupils are often unequal. | Always equal. |
| Deafness often complete and permanent. | Hardly ever permanent, or attended with signs of disorganization of the ear. |
| Duration very indefinite ; but generally from four to seven days. | Duration from twelve to fourteen days. |
| Relapses are common. | Relapses are rare. |
| The blood is often highly fibrinous. | The blood is never fibrinous. |
| The lesions, unless in the most rapid cases, consist of a fibrinous or purulent exudation in the meshes of the cerebro-spinal pia mater, | There are no inflammatory lesions whatever. |
| Mortality from 20 to 75 per cent. | Mortality from 8 to 40 per cent. |

Our space prevents our answering but one other question asked by those who have not yet seen, but every day expect to meet the disease. What *treatment* do you find most satisfactory? The following suggestions copied from an essay by J. Netten Radcliffe published in a work upon diseases of the spine and nerves, will furnish in brief as satisfactory answer as can be given.

"*Prophylactic.*—Ignorance of the true etiology of the disease limits our preventive efforts to general sanitary measures, applicable to all epidemic diseases, for the purification of houses and localities. Mr. J. Simon, recording the conditions under which the disease has prevailed, writes: "I am strongly of opinion that the best sanitary precaution which in the present state of knowledge can be taken against the disease, must consist in care for the ventilation of dwellings". He adds, however, "that in some cases, according to local reports, the distribution of an epidemic has very decidedly not been governed by conditions of overcrowding and ill-ventilation." Dr. B. W. Richardson's suggestion as to the cause of the disease should lead to the careful microscopic examination of all bread stuffs and farinaceous preparations in use among families and communities where the disease breaks out, and the disuse of such as may be of doubtful character.*

*Curative.*—The treatment of epidemic cerebro-spinal meningitis is as unsatisfactory as that of cholera. The evidence of the course of the disease having been beneficially affected in any outbreak by the administration of medicine is very doubtful. The too common rapid progress of the malady to death, as in cholera, and the nature of the lesions determining death, necessarily set at naught efforts to control it; medicine not being guilty either of inaptitude or inactivity. The control of this disease, as of cholera or trichiniasis, is a question of preventive rather than curative treatment, and must depend upon the discovery and limitation of its cause. In the earlier outbreaks, epidemic cerebro-spinal meningitis was treated as an acute inflammatory affection, by bleeding and purgatives, with the general result that the fatality of the malady was probably invariably augmented. During the recent outbreak in Philadelphia, it was found that, in the more asthenic cases, cupping the nape of the neck was "of essential service in mitigating, and generally, indeed, in wholly removing the neuralgic pains which form so prominent and so severe a symptom in many cases of the disease" (STILLE). When the state of the patient forbade the abstraction of blood, dry-cupping used in the same locality afforded signal relief, and rendered the effects of vesication more prompt and complete. This was the experience of one of the least fatal outbreaks recorded. The experience of the majority of epidemics has been against any bloodletting, local or general. The deduction to be derived as to depletion from the general state of the circulation and the results of practice entirely coincide. For, as a rule, the pulse from the very outset contraindicates the withdrawal of blood; and, if in any case it should seem from the general symptoms that depletion might exercise some control over the central mischief, a thoughtful regard should be given to the future. The application of *cold* to the head and spine, either by means of ice or a freezing mixture, in Esmarch's India-rubber bags, is not open to the same objection as bloodletting, and has furnished by far the most satisfactory results of all direct treatment of the acute cerebro-spinal symptoms. In its use care should be taken not to prolong the application so as to depress or increase the depression already existing of the whole system. When the acute nervous symptoms are accompanied by marked prostration, it is advisable during the application of the ice to swathe the limbs in hot flannels, pack the legs and thighs with hot-water bottles, or bags filled with hot sand or salt,

---

* *Diseased grain.*—Dr. B. W. Richardson has suggested that epidemic cerebro-spinal meningitis may possibly arise from the consumption of diseased grain, after the manner of ergotism, and perhaps acrodynia. He thinks that the probabilities are altogether in favour of the suggestion, that "the cause, in fact, is a diseased grain, or fungus, contained in some kinds of flour out of which the bread-stuffs are made. This fungus may not be present in large quantities, and many persons may eat of the food without getting a poisonous part; but one will get it out of a number, and this without any communication beyond the breaking of bread together: this disease may occur in one member of a family, leaving the rest free, and in this irregular way it may be distributed, in an epidemic form, over a large surface of the country." He adds, "If my hypothesis, as regards cause, be correct, there is little danger of the disorder extending widely in this country; for of our cereals used as food, nearly the whole of the population now select wheat, and our wheat generally is selected for the market with great judgement aad circumspection. Any cases, therefore, that might occur would be isolated, and would be easily traced out and prevented." This suggestion opens out an altogether new field of inquiry respecting the origin of the disease, and it demands active and thoughtful consideration in subsequent outbreaks. Dr. H. Day, of Stafford, has endeavoured, by experiments on the lower animals, to obtain some light on the subject. He fed three rabbits with unsound grain (wheat, oats, ergot of rye, and mouldy bread) with this result: In all the animals a spasmodic affection was produced, and in two inflammatory changes in the right eye, proceeding in one case to ulceration of the cornea, and evacuation of the contents of the globe One of the rabbits died on the eighth day, the other two were killed on the twelfth day, and in all more or less congestion of the membrances of the spinal cord was found on dissection.

The sum of our knowledge of the etiology of epidemic cerebro-spinal meningitis is this; that the clue to its explanation has not been discovered.

and cover the abdomen with thick layers of flannel or cotton-wool. From the very outset of the disease, care should be taken to economize the temperature of the body, and anticipate its fall; and in cases characterized by collapse, or much vital depression, the application of external heat in the manner just suggested is a cardinal point of treatment. Of medicaments directly addressed to the nervous symptoms, *opium* is the most valuable. It is especially indicated when there is much restless and delirium, sleeplessness, hyperæsthesia, and painful spasm. *Morphia* is the best form of administration, and subcutaneous injection perhaps the best mode. The drug should be given in decided and frequently repeated doses, and carefully watched. Stille says of its use during the recent outbreak in Philadelphia: "We were in the habit of giving one grain of opium every hour, in very severe, and every two hours in moderately severe cases, and in no instance was produced either narcotism, or even an approach to that condition. Under the influence of the medicine the pain and spasm subsided, the skin grew warmer, and the pulse fuller, and the entire condition of the patient more hopeful. It seemed probable, however, that the full benefit of the opium treatment could be received by those only who were subjected to it in the early stages of the attack. Direct experience is here in pefect accord with the expectation which a knowledge of the pathological processes involved in the disease would naturally suggest."

A Committee of the American Medical Association has reported favorably of the *sulphate of quinia* in large doses, given at the very beginning of the disease. In some instances the drug seemed to abort the attack. The committee speaks also of the favourable results reported from the combined use sf *ergot* and *chloride of iron*. Some American physicians have given *ergot* in combination with *belladonna*, and *belladonna* in combination with *quinine*, but with equivocal benefit. *Mercurials* have been freely used, particularly in the form of *calomel*, but their effect has been most questionable, except as purgatives. Their indiscriminate use is to be utterly condemned, and their use at all to be discountenanced. A host of other medicaments have been made use of, of which it is requisite to note only *iodide of potassium*, *bromide of potassium*, and *arsenite of potash*. The circumstances under which the two former drugs have been used, and are most likely to prove beneficial, will suggest themselves to the practitioner. It does not appear that any decided good has arisen from their administration. In protracted cases of convalescence the arsenite of potash may prove a valuable remedy.

Of the general treatment of the patient the hot bath [102°–103°] is, when practicable, the most important. This should be followed, as recommended by the Committee of the American Medical Association, by friction with warm oil of turpentine. The *regimen* should be generous and nutritious from the beginning of the disease. In the acute stages soup of some kind or other, or milk, is needed; and as soon as appetite returns, *solid* viands of any disgestible character must be given. In the graver cases where there is much restlessness and spasm or stupor, and food cannot be given by the mouth from the patient's refusal or inabillity to swallow, an attempt should be made to administer it by the rectum: when there is much thirst, the patient's fierce desire for drinks may be freely indulged. The state of the pulse is the principal guide to the use of *stimulants*. Their administration as a special remedy independently of the indications which generally govern their use has not been followed by good results; but they are called for when the condition of the pulse and the aspect of the patient show manifest flagging of the vital power. The *sequelæ* of the disease must be treated on ordinary principles.

Too frequently the state of the patient as to delirium, spasm, and irritability of the stomach prevents all internal treatment whether by medicine or food, and limits the efforts of the physician to external measures, restricting even their application. To this unhappy combination of unfortunate and uncontrollable conditions may reasonably be attributed to some extent the inefficacy of treatment."

Much might be added of personal experience, but for the present this brief outline will suffice. At present, in Buffalo, the severity of attack in new cases is less than at first outbreak of the disease.

